# Survival Prediction in Septic ICU Patients: Integrating Lactate and Vasopressor Use with Established Severity Scores

**DOI:** 10.3390/diseases14010011

**Published:** 2025-12-29

**Authors:** Celia María Curieses Andrés, Maria del Pilar Rodriguez del Tio, Ana María Bueno Gonzalez, Mercedes Artola Blanco, Silvia Medina Díez, Amanda Francisco Amador, Elena Bustamante Munguira, José M. Pérez de la Lastra

**Affiliations:** 1 Hospital Clínico Universitario de Valladolid, Avenida de Ramón y Cajal, 3, 47003 Valladolid, Spain; cmcuriesesa@saludcastillayleon.es (C.M.C.A.); ambueno@saludcastillayleon.es (A.M.B.G.); martola@saludcastillayleon.es (M.A.B.); smedinad@saludcastillayleon.es (S.M.D.); afrancisco@saludcastillayleon.es (A.F.A.); 2 Faculty of Sciences, Department of Statistics and Operational Research Office A225, University of Valladolid, P.º de Belén, 7, 47011 Valladolid, Spain; pilar.rodriguez@uva.es; 3 Institute of Natural Products and Agrobiology, CSIC-Spanish Research Council, Avda. Astrofísico Fco. Sánchez, 3, 38206 La Laguna, Spain

**Keywords:** ICU, APACHE II, SOFA, lactate, vasopressors

## Abstract

Background: Accurate prediction of survival in septic patients remains a major challenge in intensive care medicine. Established severity scores such as the Acute Physiology and Chronic Health Evaluation II (APACHE II) and the Sequential Organ Failure Assessment (SOFA) are widely used to estimate prognosis, while biochemical markers such as serum lactate may provide complementary information. However, the prognostic interplay between these scores, lactate dynamics, vasopressor requirement, and infection focus has not been fully elucidated in septic populations. Methods: We conducted a retrospective observational study of 146 adult patients with sepsis admitted to the intensive care unit (ICU) of the Hospital Clínico Universitario de Valladolid (HCUV), Spain, between 2022 and 2024. Demographic data, APACHE II and SOFA scores at admission, lactate levels at admission and 24 h, albumin, and procalcitonin were recorded. Vasopressor use (categorized by intensity) and infection focus (urinary vs. non-urinary) were documented. The primary outcome was ICU mortality. Correlation analyses (Pearson or Spearman as appropriate) were performed separately for urinary and non-urinary subgroups. Multivariable logistic regression models were constructed using APACHE II, SOFA, log-transformed lactate at 24 h, vasopressor use, and urinary focus as predictors. Model performance was assessed using Nagelkerke R^2^, area under the ROC curve (AUC), and classification accuracy. Results: ICU mortality was 23.3%. APACHE II (OR 1.092; *p* = 0.004) and SOFA (OR 1.185; *p* = 0.023) were independent predictors of ICU mortality, while log-transformed lactate at 24 h showed a positive trend (OR 1.920; *p* = 0.066). The addition of urinary focus (protective effect, OR 0.19; *p* = 0.035) and vasopressor requirement (OR 2.20; *p* = 0.04) modestly improved model discrimination (Nagelkerke R^2^ = 0.395). ROC analyses showed AUCs of 0.800 for APACHE + SOFA + log-lactate, 0.824 for the vasopressor model, and 0.833 for the urinary focus model. The best-performing models achieved >85% overall accuracy, with specificity consistently above 95%. Conclusions: In septic ICU patients, APACHE II and SOFA scores remain independent predictors of ICU mortality, and lactate at 24 h adds prognostic value—particularly in non-urinary infections. Vasopressor requirement and infection focus modestly improved model discrimination, underscoring their clinical relevance. These findings suggest that integrating severity scores with selected metabolic and clinical variables may modestly refine survival prediction in septic patients.

## 1. Introduction

Survival prediction in septic ICU patients remains a fundamental challenge in intensive care medicine [1]. Intensive Care Units (ICUs) care for patients with life-threatening conditions in which early identification of those at highest risk of death is essential to guide clinical decision-making, allocate resources, and improve outcomes [2]. Prognostic assessment tools, particularly those combining clinical scores with biochemical biomarkers, have gained increasing relevance for stratifying risk and informing both bedside and research decisions [3].

Among the most widely used severity scoring systems are the Acute Physiology and Chronic Health Evaluation II (APACHE II) and the Sequential Organ Failure Assessment (SOFA) [4,5]. APACHE II, introduced in 1985, is a composite score that incorporates acute physiological derangements, age, and chronic health status to predict mortality risk in ICU patients [6]. It has been validated in multiple populations and remains a cornerstone of outcome prediction in critical care [7]. The SOFA score, first described in the 1990s, focuses on the extent of organ dysfunction across six systems: respiratory, cardiovascular, hepatic, coagulation, renal, and neurological [8]. Originally developed for patients with sepsis, SOFA is now widely used for prognostic evaluation in all ICU patients, and it has been incorporated into the Sepsis-3 definitions as a key tool for identifying organ dysfunction [9].

In parallel to these scoring systems, several biochemical markers have emerged as potential prognostic indicators in critical illness. Serum lactate is of particular interest because it reflects the balance between oxygen delivery and consumption and serves as a marker of tissue hypoxia, impaired clearance, or metabolic stress [10]. Elevated lactate levels have consistently been associated with increased mortality across sepsis cohorts and other critical care populations. Both initial lactate concentrations and lactate kinetics (changes over time) have been shown to provide prognostic information, and lactate clearance is often used as a surrogate endpoint in resuscitation protocols [11,12,13,14].

Albumin, a marker of nutritional status and systemic inflammation, has also been associated with ICU outcomes. Hypoalbuminemia can reflect chronic comorbidities, acute inflammatory states, or capillary leak syndromes, and it has been linked to worse outcomes in septic and non-septic patients alike. The ratio of lactate to albumin has been proposed as an integrated prognostic index, potentially capturing both metabolic stress and nutritional/inflammatory status [15,16,17].

Procalcitonin, a peptide precursor of calcitonin, is released in response to bacterial infections and systemic inflammation. Its role as a prognostic marker remains debated, but in the context of sepsis, higher levels have been correlated with disease severity and mortality. Serial procalcitonin measurements may provide additional information beyond single values, especially when combined with other parameters [18,19].

While each of these variables—APACHE II, SOFA, lactate, albumin, and procalcitonin—has been studied independently, fewer investigations have integrated them into a single multivariable model to predict ICU survival in septic patients. Moreover, the influence of infection focus on the prognostic value of these markers remains poorly characterized. Different infection sites may elicit distinct pathophysiological responses: for example, urinary tract infections may produce lower systemic inflammatory and metabolic derangements compared to abdominal or respiratory infections. Understanding whether biomarker relationships vary by infection site could help tailor prognostic models and interventions [20,21].

Retrospective and prospective studies have suggested that the association between lactate and other biomarkers may differ depending on the underlying cause of critical illness. In septic shock, lactate elevations may result from both tissue hypoxia and non-hypoxic mechanisms such as mitochondrial dysfunction or adrenergic stimulation [22,23]. In urinary infections, the extent of tissue hypoperfusion may be less pronounced than in intra-abdominal or pulmonary sepsis, potentially altering the relationship between lactate, inflammatory markers, and outcomes. For example, an inverse correlation between lactate and albumin might be expected in high-inflammatory states with capillary leakage, whereas in more localized infections such as urinary sepsis, this association could be weaker or absent [24].

The integration of severity scores and biochemical markers into multivariable prediction models could improve prognostic accuracy compared to individual parameters. Logistic regression and other statistical approaches allow for simultaneous evaluation of multiple predictors, estimation of their independent contributions, and assessment of overall model performance through metrics such as the Nagelkerke R^2^ and classification accuracy [25]. However, model performance may vary depending on patient subgroups, such as those defined by infection focus, and it is unclear whether adding biochemical markers meaningfully improves the predictive power of established scores in all septic populations [26,27].

In addition to severity scores and biochemical markers, the requirement for vasopressor support is a critical indicator of hemodynamic instability and poor prognosis in ICU patients. Vasopressors, such as norepinephrine and other vasoactive amines, are often necessary in septic shock and their use has been consistently associated with increased mortality. They can reflect both the severity of circulatory failure and the intensity of supportive therapy. Integrating vasopressor requirement into prognostic models may therefore improve risk stratification, especially when combined with APACHE II, SOFA, and lactate dynamics [28,29].

The present study aims to evaluate the prognostic value of APACHE II, SOFA, serum lactate (at 24 h), and vasopressor use for predicting ICU mortality in septic patients admitted to the Intensive Care Unit of the Hospital Clínico Universitario de Valladolid (HCUV), Spain, between 2022 and 2024. We also explore the correlations between lactate, albumin, and procalcitonin stratified by infection focus (urinary vs. non-urinary) to determine whether biomarker relationships differ by site of infection.

We hypothesize that (1) APACHE II and SOFA scores will be independent predictors of ICU mortality; (2) lactate and vasopressor requirement will provide additional prognostic information; and (3) the correlation between lactate and albumin will be present in non-urinary infections but absent in urinary infections. By examining both integrated prognostic modeling and focus-specific biomarker relationships, this study seeks to contribute to the refinement of survival prediction in sepsis and to inform more personalized approaches to patient monitoring and management.

## 2. Materials and Methods

### 2.1. Study Objective

**Figure 1:**
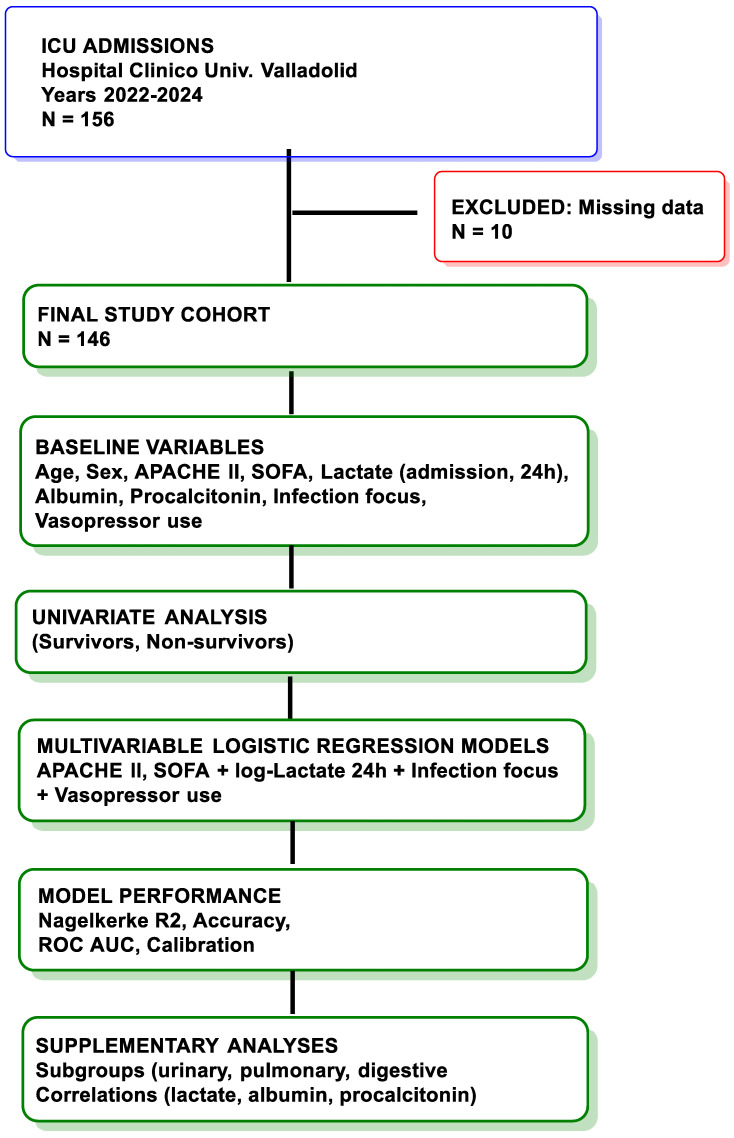
Flow diagram of patient inclusion. Of 156 patients admitted to the ICU during the study period, 10 were excluded due to missing key variables. The final cohort included 146 patients with sepsis or septic shock, of whom 112 survived and 34 died during their ICU stay.

The primary objective of this study (Figure 1) was to evaluate the predictive value of APACHE II and SOFA scores, together with serum lactate at 24 h (including its logarithmic transformation), for ICU mortality among adult patients with sepsis or septic shock admitted to the ICU. Secondary objectives were to assess whether biochemical markers (albumin, procalcitonin, lactate-to-albumin ratio) and clinical variables (vasopressor use and infection focus) provided incremental prognostic value. Model performance was evaluated using Nagelkerke R^2^, classification accuracy, sensitivity, specificity, predictive values, and the area under the ROC curve (AUC).

### 2.2. Study Design and Setting

This was a retrospective observational cohort study conducted in the Intensive Care Unit (ICU) of the Hospital Clínico Universitario de Valladolid (HCUV), a tertiary care center in Valladolid, Spain. The study period extended from January 2022 to March 2024. The ICU is a 21-bed mixed medical-surgical unit providing comprehensive care for critically ill adult patients.

### 2.3. Patient Population

All consecutive adult patients (≥18 years) admitted during the study period who fulfilled the Sepsis-3 criteria for sepsis or septic shock [20] were screened. Patients without complete records for APACHE II, SOFA, or lactate were excluded. The final cohort included 146 septic patients.

### 2.4. Data Collection

Data were obtained from electronic medical records and included: Demographics: age, sex. Severity scores: APACHE II and SOFA at admission. Biomarkers: lactate at admission and 24 h, albumin, and procalcitonin. Derived markers: log-transformed lactate at 24 h, lactate/albumin ratio. Clinical variables: vasopressor use, infection focus (urinary vs. non-urinary). Outcome: ICU survival (discharge from ICU vs. death in ICU). Vasopressor use (amines) was defined as administration of vasoactive amines (e.g., norepinephrine, dopamine, epinephrine, vasopressin) during the first 24 h of ICU stay. Vasopressor support was categorized according to standardized dosing thresholds as follows: none, low-dose (<0.3 µg/kg/min), medium-dose (0.3–0.6 µg/kg/min), high-dose (>0.6 µg/kg/min), or multiple vasopressors used concurrently (+VP). In some logistic regression models, vasopressor support was also dichotomized as any vs. none to improve model stability. Procalcitonin was available as two variables in the source dataset (PROCALT at admission and PROCALT at 24 h). For consistency, we treated procalcitonin as an exploratory biomarker and report its univariable discrimination in the Supplementary Figure S1. Lactate at 24 h was additionally log-transformed for modeling and correlation analyses, as specified a priori.

### 2.5. Statistical Analysis

Continuous variables were summarized as mean ± standard deviation or median (interquartile range), depending on distribution, and categorical variables as counts and percentages. Group comparisons between survivors and non-survivors were performed using Student’s *t* test or Mann–Whitney U test for continuous variables and the chi-square test for categorical variables.

Multivariable logistic regression was used to identify independent predictors of ICU mortality. Candidate predictors included APACHE II, SOFA, log-transformed lactate at 24 h, vasopressor use, and infection focus. Vasopressor support was entered either as a categorical variable (no, low, medium, high, multiple) or dichotomized (any vs. none) in sensitivity analyses to reduce instability from small subgroups. Infection focus was modeled as a binary variable (urinary vs. non-urinary). Biomarkers such as albumin, procalcitonin, and the lactate-to-albumin ratio were also explored but were not retained in the final models when non-significant.

Model performance was assessed using −2 log-likelihood, Cox & Snell and Nagelkerke R^2^ statistics, and the Wald test for variable significance. Discriminatory capacity was evaluated by receiver operating characteristic (ROC) curve analysis, with area under the curve (AUC) reported for individual predictors and combined models. Sensitivity, specificity, predictive values, and overall accuracy were derived from classification tables at a cut-off of 0.500. Calibration was visually assessed with calibration plots. All analyses were conducted using SPSS Statistics (IBM Corp., version 29, New York, NY, USA). A two-tailed *p* < 0.05 was considered statistically significant.

### 2.6. Ethical Considerations

This study was conducted in accordance with the ethical standards of the institutional and national research committees and with the 1964 Helsinki declaration and its later amendments. Formal approval by the Ethics Committee of the Hospital Clínico Universitario de Valladolid was not required, as this was a retrospective study using fully anonymized data and posed no risk to patients. The requirement for informed consent was therefore waived.

## 3. Results

### 3.1. Study Population

A total of 146 adult patients with sepsis or septic shock admitted to the ICU of the Hospital Clínico Universitario de Valladolid (HCUV) between January 2022 and March 2024 were included in the analysis. All patients fulfilled Sepsis-3 criteria at the time of ICU admission.

The overall ICU mortality was 23.3% (n = 34 deaths). The median age was 68 years, and 60% of patients were male. Non-survivors tended to be younger, had significantly higher severity scores (APACHE II and SOFA), and exhibited higher serum lactate concentrations at 24 h compared to survivors. Albumin and procalcitonin levels did not significantly differ between groups. Baseline characteristics of septic ICU patients stratified by survival status are shown in Table 1 (see also Supplementary Table S1 for the complete dataset). Urinary tract infections were more frequent among survivors, while vasopressor use was more frequent and at higher intensity among non-survivors (Table 1).

### 3.2. Univariable Predictors

**Figure 2:**
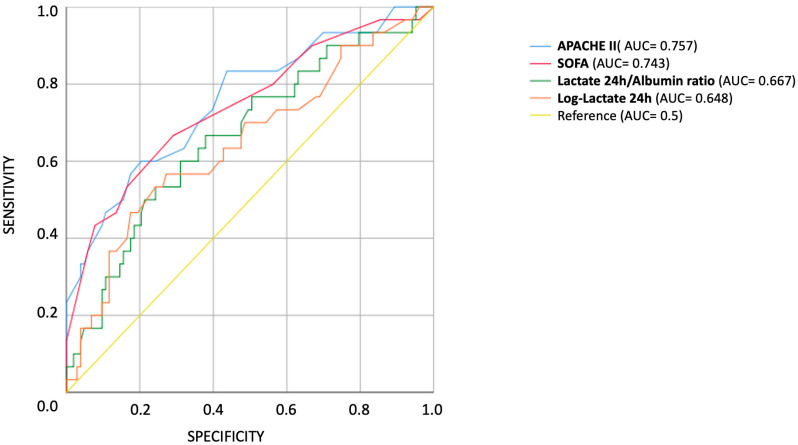
Receiver operating characteristic (ROC) curves for exploratory biomarkers in septic ICU patients. ROC curves depict the ability of procalcitonin, admission lymphocyte percentage, lactate at 24 h, and lactate-to-albumin ratio to predict ICU mortality. The analysis demonstrates modest predictive ability for lactate at 24 h and LAR, while procalcitonin and lymphocyte percentage were not predictive of ICU mortality. The diagonal line represents the line of no discrimination (AUC = 0.5).

In univariable ROC analyses restricted to septic patients, APACHE II (AUC = 0.757) and SOFA (AUC = 0.743) demonstrated good discriminatory ability for ICU mortality, whereas log-transformed lactate at 24 h (AUC = 0.648) and the lactate-to-albumin ratio (AUC = 0.667) showed only modest predictive value (Figure 2). Procalcitonin (AUC = 0.510) and admission lymphocyte percentage (AUC = 0.552) were not predictive of ICU mortality. Full AUC values with 95% confidence intervals and predictor variables are provided in Supplementary Tables S2 and S3, respectively. Exploratory ROC curves for additional biomarkers are shown in Supplementary Figure S1.

### 3.3. Correlations Between Biomarkers

In patients without a urinary infection focus, log-transformed lactate at 24 h showed a significant positive correlation with log-procalcitonin at 24 h (ρ = 0.353, *p* < 0.001), while its correlation with albumin was weak and not statistically significant (ρ = −0.160, *p* = 0.11). In patients with a urinary infection focus, log-lactate at 24 h was also significantly correlated with log-procalcitonin (ρ = 0.559, *p* < 0.001), whereas its correlation with albumin was negligible and not significant (ρ = −0.056, *p* > 0.3). These findings indicate that lactate dynamics were associated with inflammatory status (procalcitonin) but not with nutritional/inflammatory status (albumin), regardless of infection focus. Scatterplots are shown in Supplementary Figure S2.

### 3.4. Multivariable Logistic Regression Models

We constructed a series of multivariable logistic regression models to assess the independent prognostic value of severity scores, lactate, infection focus, and vasopressor use for ICU mortality among septic patients (Table 2). In the baseline model (Model A), which included only APACHE II and SOFA scores at ICU admission, both were independent predictors of ICU mortality (APACHE II: OR 1.106, *p* = 0.001; SOFA: OR 1.185, *p* = 0.020), with a Nagelkerke R^2^ of 0.324 and correct classification of 84.5% of patients.

When log-transformed lactate at 24 h was added (Model B), model performance improved slightly (Nagelkerke R^2^ = 0.348, accuracy 84.9%). Log-lactate showed a positive trend toward predicting ICU mortality (OR 1.920, *p* = 0.066), although it did not reach conventional statistical significance.

Replacing log-lactate with the lactate-to-albumin ratio (Model C) yielded similar discrimination (Nagelkerke R^2^ = 0.329, accuracy 85.0%), but the ratio was not an independent predictor (OR 1.411, *p* = 0.212). In Model D, incorporating infection focus (urinary vs. non-urinary) alongside APACHE II, SOFA, and log-lactate, urinary infection was independently associated with lower risk of mortality (OR 0.188, *p* = 0.035). This model demonstrated improved fit (Nagelkerke R^2^ = 0.395, accuracy 85.6%).

Finally, Model E integrated vasopressor category as an additional variable. This model achieved the highest explanatory power (Nagelkerke R^2^ = 0.448, overall accuracy 89.6%). Escalating vasopressor requirement was strongly associated with increased mortality, although confidence intervals were wide due to small subgroup sizes.

Taken together, these results indicate that APACHE II and SOFA remain robust independent predictors of ICU mortality in septic patients, while lactate (log-transformed), infection focus, and vasopressor requirement add incremental prognostic value.

## 4. Discussion

In this retrospective cohort of adult septic patients admitted to a tertiary care ICU, we found that established severity scores (APACHE II and SOFA) remained independent predictors of ICU mortality, consistent with their longstanding validation across diverse ICU populations [20,26,27,30]. Importantly, lactate at 24 h provided incremental prognostic information, with log-transformed lactate showing a trend toward independent prediction. This finding aligns with prior studies demonstrating the prognostic value of lactate kinetics and 24 h lactate levels as indicators of tissue hypoxia, metabolic stress, and inadequate resuscitation [11,12,13].

Our results extend previous evidence by demonstrating that the integration of clinical severity scores and biochemical markers yields slightly higher prognostic performance compared with individual parameters. The combined model achieved good discrimination (AUC 0.800), further improved when urinary infection focus and vasopressor use were incorporated (AUC 0.833). Strategies using multifactorial models that integrate global disease severity markers (like APACHE II or SOFA scores) with metrics of treatment intensity (such as lactate levels and vasopressor requirements) can enhance the accuracy of ICU mortality prediction. A study by Hayashi et al. (2020) demonstrated that incorporating maximal arterial lactate at 24 h into an APACHE III-based model increased its AUC from 0.771 to 0.815 for predicting ICU mortality in unselected ICU patients [31].

Our sequential modeling approach further illustrates this stepwise improvement. Starting from a baseline model incorporating APACHE II and SOFA (Model A), we observed incremental gains in model fit upon adding log-transformed lactate at 24 h (Model B). Substituting in the lactate-to-albumin ratio (Model C) yielded similar performance, although the lactate-to-albumin ratio did not contribute significantly after adjustment for APACHE II and SOFA. Incorporating infection focus (Model D) improved calibration and revealed that urinary infections were associated with lower risk. Finally, the best-performing model in our dataset occurred when vasopressor requirement was included (Model E), resulting in the highest classification accuracy and Nagelkerke R^2^. These findings underscore that models combining severity scores with metabolic and treatment-related variables offer superior survival prediction in septic ICU patients. Similar stepwise improvements have been reported in sepsis cohorts, where integrating lactate trends into the SOFA framework significantly improved discrimination compared to SOFA alone (SOFA AUC 0.656 vs. Lac–SOFA day 3 AUC 0.797; *p* < 0.001) [32].

We also explored whether the prognostic interplay of biomarkers varied according to infection focus. In non-urinary infections, we did not observe statistically significant correlations between albumin and log-transformed lactate at 24 h, but log-procalcitonin at 24 h showed a moderate positive correlation with log-lactate. A similar pattern was seen in urinary infections, where albumin and log-lactate were not correlated, while the association between log-procalcitonin and log-lactate was stronger. These findings suggest that, within this septic ICU population, lactate dynamics were more closely related to inflammatory activity than to nutritional status, regardless of infection focus. Such focus-specific differences have rarely been explored in prognostic studies. Clinical observations have shown that the lactate-to-albumin ratio (LAR) behaves differently depending on infection site, with variable predictive performance across disease contexts like sepsis with or without hepatic involvement [33,34]. These findings support the notion that predictive validity of biomarkers is context-dependent—emphasizing the importance of tailoring prognostic models to underlying pathophysiology and infection origin.

By defining vasopressor intensity according to standardized dosing thresholds, our study provides a clinically meaningful stratification of circulatory support requirements, reinforcing the role of escalating vasopressor use as a marker of poor prognosis, consistent with the established relationship between increasing hemodynamic support and ICU mortality in septic shock and other critical illness states [28,29,35]. However, although ROC analyses confirmed good discrimination for models including vasopressor use (AUC~0.82), categorical coding of vasopressor intensity did not independently enhance multivariable models once APACHE II, SOFA, and lactate were included. This suggests that while vasopressor use is an important bedside marker of circulatory failure, its prognostic contribution may already be largely captured by established severity scores and metabolic markers, thereby offering only modest incremental benefit [36].

The lactate-to-albumin ratio (LAR) has been proposed as a composite biomarker integrating metabolic and nutritional status. Prior large-scale retrospective studies in MIMIC-III and eICU cohorts demonstrated that LAR outperformed lactate alone in predicting ICU mortality, particularly in patients with hepatic or renal dysfunction [16,17]. Our results only partially support previous observations on the prognostic role of the lactate-to-albumin ratio (LAR). In our cohort of septic ICU patients, LAR demonstrated only modest discriminatory ability (AUC = 0.656) and did not add significant value once APACHE II and SOFA were incorporated into multivariable models. These findings contrast with reports in other populations. For example, Zhao et al. observed in polytrauma patients that combining APACHE II, Injury Severity Score (ISS), and LAR at 48 h markedly improved prognostic accuracy (AUC = 0.968), with each variable remaining independently predictive after logistic regression [37]. Similarly, studies in sepsis have shown that LAR can outperform lactate alone in predicting short- and mid-term mortality [38,39]. Differences between our results and these prior reports may reflect the relatively small cohort size, differences in patient mix, and the lower prevalence of severe hepatic dysfunction in our population, conditions where LAR is thought to have greater discriminatory ability.

Our focus-specific analyses revealed that in patients with digestive infections, both serum lactate and LAR showed strong predictive performance (AUC > 0.80), whereas in urinary and pulmonary infections, these markers performed poorly. To date, few studies have examined biomarker performance stratified by infection site. However, there is evidence that the prognostic utility of lactate-related metrics depends on the infection focus—for example, lactate clearance after fluid resuscitation showed varying predictive ability based on whether the source was pulmonary or non-pulmonary [40]. Our findings further emphasize that prognostic models are context-dependent. The clinical and pathophysiological variability across infection sites underscores the importance of tailoring predictive algorithms to underlying mechanisms, rather than relying on one-size-fits-all biomarkers. It is noteworthy that digestive infections (representing hollow viscus involvement) showed the strongest predictive value for lactate and LAR, in contrast to urinary or pulmonary infections. This supports the hypothesis that hollow viscus infections may share distinct pathophysiological mechanisms that amplify metabolic and inflammatory stress. Although consistent with prior clinical observations, our sample size for digestive cases was modest, and these findings warrant confirmation in larger cohorts.

Our findings are consistent with prior research highlighting the prognostic relevance of both APACHE II and SOFA. Numerous multicenter analyses have confirmed their reliability across sepsis, trauma, and mixed ICU cohorts [20,26,27,30]. However, our study adds nuance by integrating lactate at 24 h into multivariable models. Several studies have emphasized lactate clearance or kinetics as predictors of outcome [11,12,13]; we extend this evidence by showing that log-transformed lactate, while not independently significant in all models, trends toward improved predictive value when combined with severity scores.

## 5. Clinical Implications, Limitations and Future Directions

The present findings highlight that survival prediction in septic ICU patients can be strengthened by integrating established severity scores with both biochemical and clinical variables. APACHE II and SOFA remain essential foundations for prognostic assessment, while lactate at 24 h provides additional insight into metabolic stress and tissue perfusion. Vasopressor requirement, as a readily available bedside marker of circulatory failure, was independently associated with ICU mortality, and infection focus (urinary vs. non-urinary) offered further discriminatory value. Together, these variables may improve risk stratification, support discussions with patients and families, and help prioritize monitoring and therapeutic interventions. Incorporating them into clinical decision-support tools could enable more personalized management and guide allocation of critical care resources. It is important to note that the improvements observed were modest, which is consistent with prior sepsis literature and should be interpreted cautiously. This study also emphasizes the prognostic significance of vasopressor use. Beyond confirming its strong association with mortality, categorizing vasopressor intensity provided insight into the gradation of hemodynamic support and offers a pragmatic variable for integration into predictive models.

This study has several limitations. First, it was conducted at a single tertiary care center, which may limit generalizability. Second, the sample size—particularly in subgroup analyses by infection focus—was modest, and some comparisons may have been underpowered. Third, biomarker measurements were limited to admission and 24 h; more granular lactate kinetics or serial albumin and procalcitonin values may have yielded additional prognostic information. Fourth, although vasopressor use was categorized by intensity, precise dosing and duration were not standardized, and residual confounding cannot be excluded. Fifth, because data were abstracted retrospectively from clinical records, variability in measurement and documentation by different personnel could have introduced minor inaccuracies or misclassification. Sixth, we did not differentiate between deaths occurring during the ICU stay and those that occurred later during hospitalization. While in-hospital mortality remains a valid and widely used endpoint, this approach may obscure differences between early deaths related to acute critical illness and later deaths influenced by post-ICU complications or comorbidities. Finally, although we stratified analyses by infection focus, the subgroup of digestive (hollow viscus) infections was relatively small.

Future research should aim to validate these findings in larger, multicenter cohorts, incorporating standardized biomarker measurements, prospective data collection, and stratification of mortality endpoints. External validation is particularly important for subgroup analyses, such as urinary versus non-urinary infections, where sample sizes in this study were limited. Furthermore, dynamic monitoring of biomarkers and vasopressor requirements may offer greater prognostic accuracy than single time-point measurements. Incorporating these temporal trends into advanced multivariable or machine-learning models could better capture nonlinear interactions between severity scores, biomarkers, and therapeutic interventions. Finally, prospective studies are needed to determine whether embedding such models into bedside decision-support tools can improve clinical outcomes by enabling earlier recognition of high-risk patients and more personalized treatment strategies.

## 6. Conclusions

In this retrospective cohort of septic ICU patients, the APACHE II and SOFA scores were confirmed as independent predictors of ICU mortality. Serum lactate at 24 h provided additional prognostic information, particularly in non-urinary infections. Vasopressor use was strongly associated with mortality and enhanced predictive performance when incorporated into multivariable models. APACHE II and SOFA remained the strongest independent predictors of mortality. Lactate at 24 h and vasopressor use showed modest additional prognostic information, suggesting that combining severity scores with metabolic and hemodynamic variables may refine risk stratification in selected patients. These findings should be interpreted with caution and validated in larger cohorts.

These findings highlight the value of integrating clinical severity scores, metabolic biomarkers, and treatment variables into composite models to refine ICU survival prediction.

## Figures and Tables

**Table 1:** Baseline characteristics of ICU patients stratified by survival status. Continuous variables are expressed as mean ± standard deviation (SD) or median [interquartile range, IQR], and categorical variables as counts (percentages). *p* values correspond to Student’s *t* test, Mann–Whitney U test, or chi-square/Fisher’s exact test, as appropriate. Vasopressor use was categorized as none, low-dose (<0.3 µg/kg/min), medium-dose (0.3–0.6 µg/kg/min), high-dose (>0.6 µg/kg/min), or multiple vasopressors used concurrently (+VP).

Variable	ICU Survivors (n = 112)	ICU Non-Survivors (n = 34)	*p* Value
Age (years, mean ± SD)	71.0 ± 11.7	66.9 ± 13.4	0.103
Sex, male (%)	50.0%	59.0%	0.340
APACHE II (mean ± SD)	19.9 ± 7.1	29.1 ± 10.6	<0.001
SOFA score (median [IQR])	6 [5]	9 [6]	<0.001
Lactate at admission * (mmol/L, median [IQR])	1.6 [1.2]	2.4 [2.7]	0.020 *
Lactate at 24 h (mmol/L, median [IQR])	1.53 [1.10]	2.25 [2.51]	0.002
Log-lactate at 24 h (mean ± SD)	0.48 ± 0.53	0.94 ± 0.85	0.004
Albumin (g/dL, mean ± SD)	3.1 ± 0.6	3.0 ± 0.5	0.280
Procalcitonin 24 h median [IQR])	4.51 [21.86]	6.64 [17.58]	0.58
LogProcalcitonin 24 h (mean ± SD)	1.34 ± 2.34	1.52 ± 1.83	0.68
Infection focus, urinary (%)	30.0%	12.0%	0.035
Vasopressor use, any (%)	41.0%	82.0%	<0.001
Vasopressor category, n (%)			<0.001
- None	32 (28.6%)	2 (5.9%)	
- Low-dose (<0.3 µg/kg/min)	65 (58.0%)	22 (64.7%)	
- Medium-dose (0.3–0.6 µg/kg/min)	5 (4.5%)	2 (5.9%)	
- High-dose (>0.6 µg/kg/min)	4 (3.6%)	2 (5.9%)	
- Multiple vasopressors (+VP)	6 (5.4%)	6 (17.6%)	

All patients fulfilled Sepsis-3 criteria on ICU admission. The primary outcome was ICU mortality. * Lactate at admission was available for a subset of patients.

**Table 2:** Multivariable logistic regression models predicting ICU mortality in septic ICU patients. Predictors included established severity scores (APACHE II and SOFA at admission), biochemical markers (serum lactate at 24 h, log-transformed lactate, and lactate-to-albumin ratio), and clinical variables (infection focus: urinary vs. non-urinary; vasopressor use categorized by intensity). Odds ratios (OR) with 95% confidence intervals (CI) are reported. Model performance was assessed using Nagelkerke R^2^ and classification accuracy.

Model	Predictors Included	OR (95% CI)	*p* Value	Nagelkerke R^2^	Correct Classification (%)
A	APACHE II	1.106 (1.04–1.17)	0.001	0.324	84.5
	SOFA	1.185 (1.03–1.36)	0.020		
B	APACHE II	1.092 (1.03–1.16)	0.004	0.348	84.9
	SOFA	1.185 (1.02–1.38)	0.023		
	log-lactate (24 h)	1.920 (0.95–3.87)	0.066		
C	APACHE II	1.097 (1.03–1.17)	0.004	0.329	85.0
	SOFA	1.173 (1.01–1.36)	0.040		
	LAR (lactate/albumin)	1.411 (0.82–2.43)	0.212		
D	APACHE II	1.086 (1.02–1.16)	0.008	0.395	85.6
	SOFA	1.176 (1.01–1.37)	0.033		
	log-lactate (24 h)	1.952 (0.96–3.99)	0.067		
	Infection focus (urinary)	0.188 (0.04–0.88)	0.035		
E	APACHE II	1.089 (1.02–1.16)	0.010	0.448	89.6
	SOFA	1.199 (1.01–1.42)	0.037		
	log-lactate (24 h)	1.469 (0.62–3.48)	0.380		
	Vasopressor category (ref = none)	—	>0.05		

## Data Availability

The datasets generated and analyzed during the current study are not publicly available due to patient confidentiality restrictions, but de-identified data may be made available from the corresponding author upon reasonable request and with appropriate institutional approvals.

## Supplementary Materials

The following supporting information can be downloaded at: https://www.mdpi.com/article/10.3390/diseases14010011/s1, Figure S1: Receiver operating characteristic (ROC) curves for exploratory biomarkers (procalcitonin, lymphocyte percentage, lactate at 24 h, and lactate-to-albumin ratio); Figure S2: Correlation scatterplots of lactate with albumin and procalcitonin, stratified by infection focus; Table S1: Baseline characteristics of ICU survivors and non-survivors; Table S2: Univariable ROC analyses for candidate predictors of in-hospital mortality. Table S3: Coding of Predictor Variables.
