# Supplementary material for: Survival Prediction in Septic ICU Patients: Integrating Lactate and Vasopressor Use with Established Severity Scores
Source: Diseases. 2025 Dec 29;14(1):11. doi: 10.3390/diseases14010011 (PMC12839871; doi:10.3390/diseases14010011)
Supplement: Supplementary file 1 [file diseases-14-00011-s001.zip › diseases-3909067-supplementary.pdf]

Supplementary Figure S1

Receiver operating characteristic (ROC) curves for exploratory biomarkers (Procalcitonin, lymphocyte percentage, lactate at 24h, and lactate-to-albumin ratio). The analysis demonstrates modest predictive ability for lactate at 24h and LAR, while procalcitonin and lymphocyte percentage were not predictive of ICU mortality.

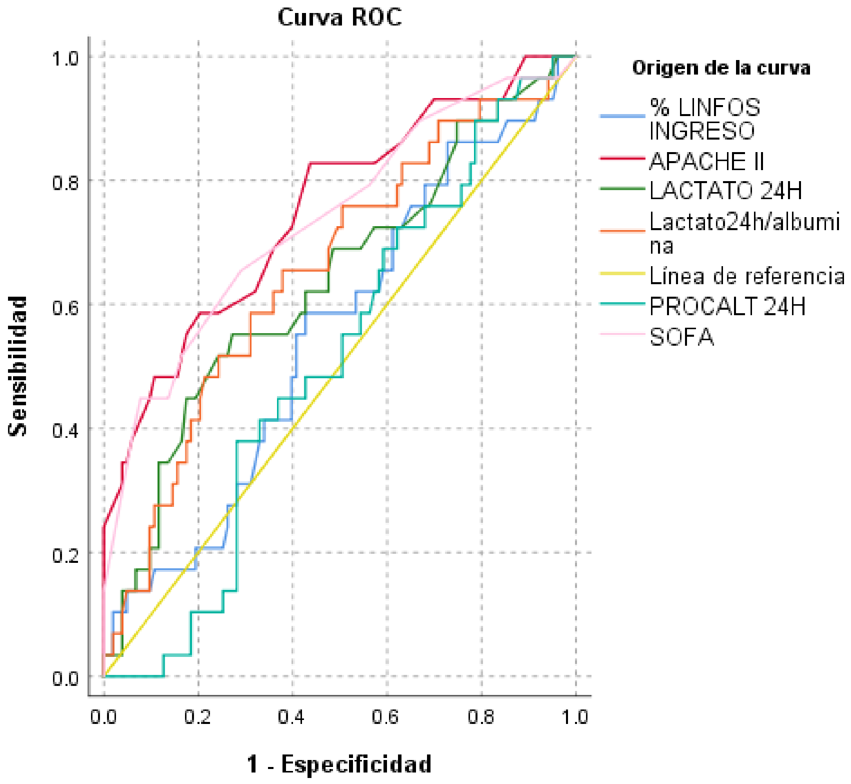

Supplementary Figure S2

Correlation scatterplots of lactate with albumin and procalcitonin, stratified by infection focus.

** For Pearson correlations, a logarithmic scale was used when it was necessary to normalize the variable (in Lactate and Procalcitonin, in Albumin it is not necessary)*

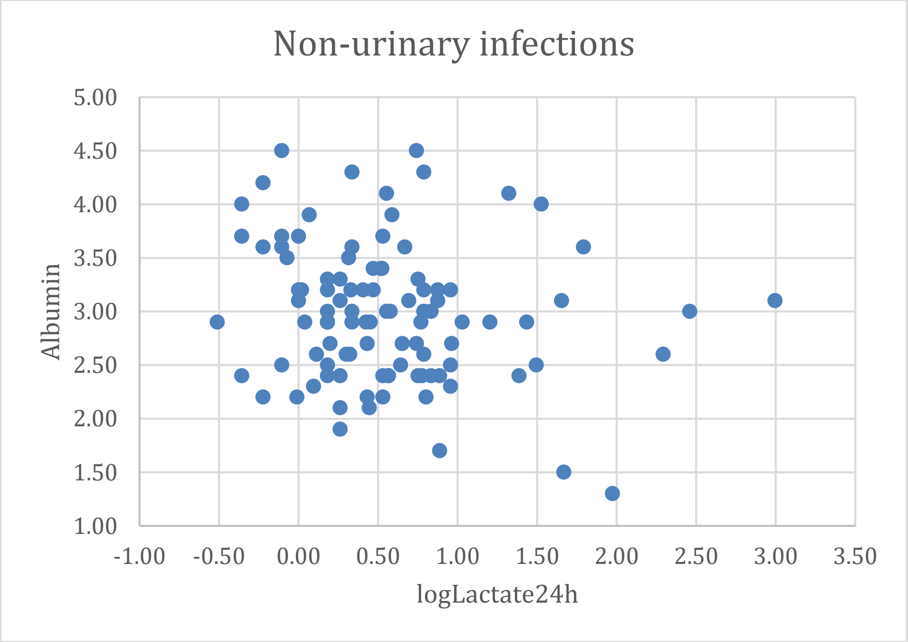

r Pearson albumine, loglactate24h= -0.175 , p-value=0.077 n=102

r Spearman= -0.160 p-value=0.11

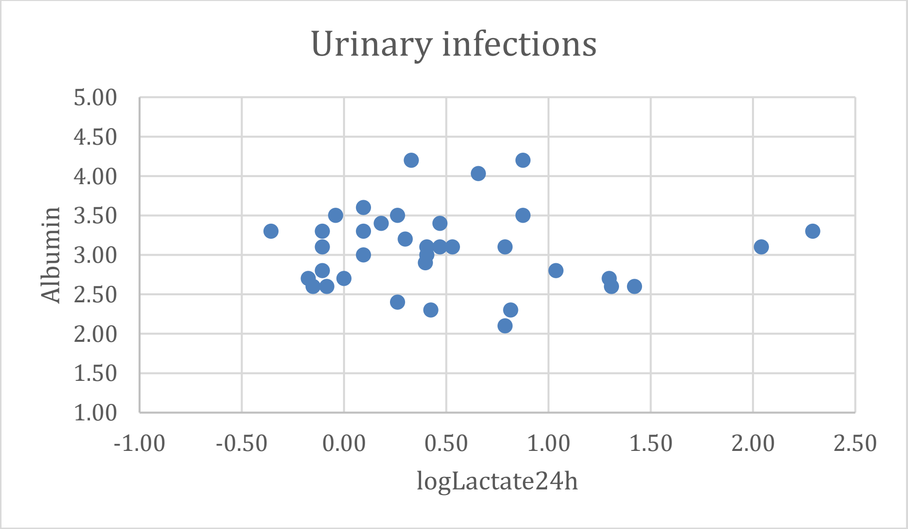

r Pearson entre albumine, loglactate24h= - 0.043 p-value=0.80 n= 36. r Spearman= -0.056 p-value>0.3

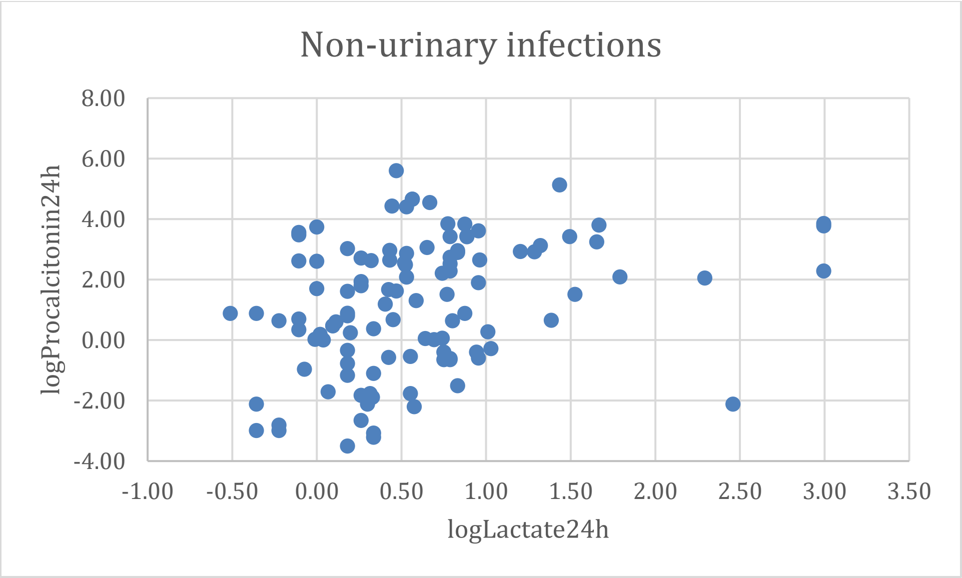

r Pearson logProcalcitonin24h, logLactate24h =0.325 p-value < 0.001 n=111

r Spearman = 0.353 p-value=0.000

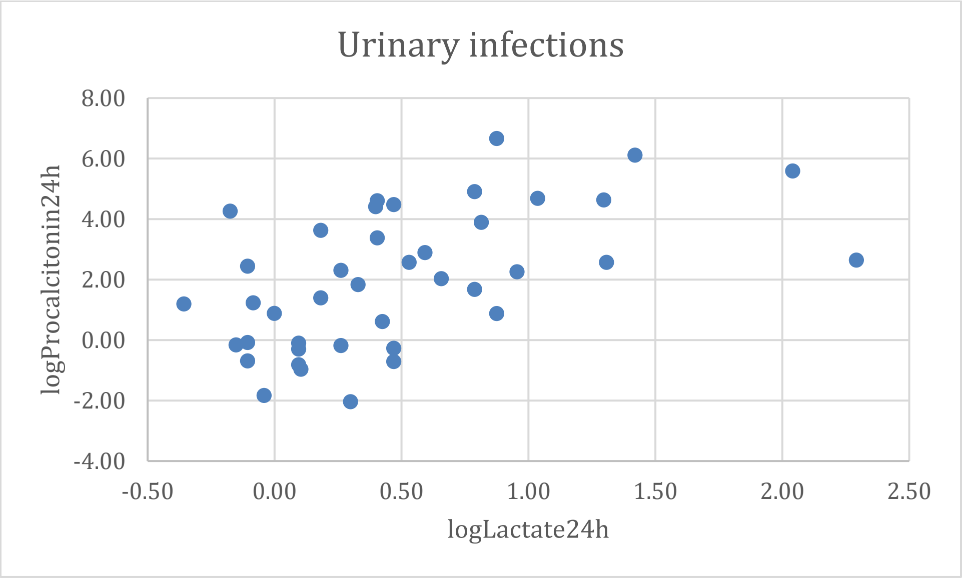

r Pearson logProcalcitonin24h, logLactate24h =0.537 p-value < 0.001 n=41

r Spearman =0.559 p-value=0.000

Table S1. Summary of Best-Performing Multivariable Models. Overview of the multivariable logistic regression models evaluated for ICU mortality prediction. For each model, the table reports the predictors included, Nagelkerke R^2, area under the receiver operating characteristic curve (AUC), overall accuracy, and relevant notes. Model E showed the highest performance within this dataset, although improvements were modest.

Model	Predictors Included	Nagelkerke R^2	AUC	Accuracy	Notes
A	APACHE II + SOFA	0.324	—	84.5%	Baseline model
B	APACHE II + SOFA + log-lactate (24h)	0.348	0.800	84.9%	Log-lactate trend (p=0.066)
D	APACHE II + SOFA + log-lactate + urinary focus	0.395	0.833	85.6%	Urinary focus protective
E	APACHE II + SOFA + log-lactate + vasopressor category	0.448	0.824	89.6%	Best-performing model

Table S2. Regression Equations for ICU Mortality Prediction. Logistic regression equations corresponding to the multivariable models described in the manuscript. For each model, the linear predictor (LP) is displayed along with the probability transformation used to estimate ICU mortality. Coefficients (β) are derived from the multivariable analyses reported in Table 2 of the main manuscript.

**LP**	β0 + β1(APACHE II) + β2(SOFA) + β3(log lactate 24h) + β4(urinary focus) + β5(vasopressor category)
Probability of ICU mortality	
P(death)	1 / (1 + e^(LP))
**Model A**	
LP_A	β0 + 1.106(APACHE II) + 1.185(SOFA)
**Model B**	
LP_B	β0 + 1.092(APACHE II) + 1.185(SOFA) + 1.920(log lactate)
**Model D**	
LP_D	β0 + 1.086(APACHE II) + 1.176(SOFA) + 1.952(log lactate) + 0.188(urinary focus)
**Model E**	
LP_E	β0 + 1.089(APACHE II) + 1.199(SOFA) + 1.469(log lactate) + β5(vasopressor category)

Table S3. Coding of Predictor Variables. Detailed coding schema for all variables included in the multivariable logistic regression analyses. This table specifies the transformation of continuous predictors, binary and categorical encodings, and definitions for vasopressor categories and infection focus to facilitate reproducibility.

APACHE II	continuous score
SOFA	continuous score
Lactate at 24h	Natural logarithm transformation (log lactate)
Infection focus	1 = urinary tract, 0 = non urinary
Vasopressor category	
0	= none
1	= low dose (<0.3 μg/kg/min)
2	= medium dose (0.3–0.6 μg/kg/min)
3	= high dose (>0.6 μg/kg/min)
4 = multiple vasopressors	
Binary vasopressor variable (sensitivity analysis):	0 = none, 1 = any vasopressor use
